# Yield of Head Imaging in Ambulatory and Hospitalized Patients With SARS-CoV-2: A Multi-Center Study of 8675 Patients

**DOI:** 10.1177/1941874420980622

**Published:** 2020-12-16

**Authors:** Melanie R. F. Greenway, Young Erben, Josephine F. Huang, Jason L. Siegel, Christopher J. Lamb, Mohammed K. Badi, Amra Sakusic, Neethu Gopal, James F. Meschia, Michelle P. Lin

**Affiliations:** 1 Department of Neurology, 23389 Mayo Clinic, Jacksonville, FL, USA; 2 Division of Vascular and Endovascular Surgery, 23389 Mayo Clinic, Jacksonville, FL, USA; 3 Department of Neurologic Surgery, 23389 Mayo Clinic, Jacksonville, FL, USA; 4 Department of Critical Care Medicine, 23389 Mayo Clinic, Jacksonville, FL, USA; 5 Department of Neurology, 23389 Mayo Clinic, Rochester, MN, USA; 6 Department of Internal Medicine, 23389 Mayo Clinic, Jacksonville, FL, USA

**Keywords:** SARS-CoV-2, COVID-19, neuroimaging, stroke, intracranial hemorrhages

## Abstract

**Background and Purpose:**

To describe the neurological and cerebrovascular findings in patients who tested positive for SARS-CoV-2 and underwent head imaging in ambulatory and inpatient settings.

**Methods:**

Consecutive patients aged ≥18 years with SARS-CoV-2 infection diagnosed or treated at Mayo Clinic sites from 3/11/2020 to 7/23/2020 with head CT or brain MRI within 30 days of SARS-CoV-2 diagnosis were included. Demographics, medical history, indication for SARS-CoV-2 testing, neurologic symptoms, indication for brain imaging, neuroimaging findings, etiology of cerebrovascular events, and hospital course were abstracted from medical records.

**Results:**

Of 8,675 patients with SARS-CoV-2, 180 (2.07%) had head imaging. Mean age of the entire cohort was 42 ± 18 years, whereas mean age of those with head imaging was 62 ± 19 years. Common indications for imaging were headache (34.4%), encephalopathy (33.4%), focal neurologic symptom (16.7%), and trauma (13.9%). While 86.1% of patients who underwent head imaging had normal exams, cerebrovascular events occurred in 18 patients (0.21% of the total cohort). Of patients with cerebrovascular events, 8 (44.5%) had acute infarct; 6 (33.3%), acute intracranial hemorrhage; 5 (2.8%), subacute infarct; and 1 (0.6%) posterior reversible encephalopathy syndrome. In the thirteen patients with ischemic stroke, 6 (46.2%) had cryptogenic stroke; 3 (23.1%), other defined causes; 2 (15.4%), small vessel stroke; 1 (7.7%), large vessel stroke; and 1 (7.7%) cardioembolic stroke.

**Conclusion:**

In ambulatory and hospitalized patients with SARS-CoV-2 infection, the rate of head imaging is low, with common indications of encephalopathy and headache. Cerebrovascular events occurred rarely, and cryptogenic stroke was the most common stroke mechanism.

## Introduction

Severe acute respiratory syndrome coronavirus 2 (SARS-CoV-2) has caused over 31 million infections worldwide, with over 961,000 deaths as of September 21, 2020. The United States accounts for more than 6.8 million infections and 199,000 deaths.^1^ Diverse neurological manifestations were originally reported in 36.4% of patients with coronavirus disease 2019 (COVID-19) in one large cohort from Wuhan, China early in the global pandemic^2^ Subsequent case reports, case series, and systematic reviews have investigated both central nervous system and peripheral nervous system manifestations of COVID-19.^2-13^ Neurologic manifestations range from nonspecific symptoms such as dizziness, headache, myalgias, and fatigue to more specific symptoms of anosmia and dysgeusia that serve as a hallmark of the disease.^14,15^ Acute ischemic stroke as a manifestation of COVID-19 has emerged as a severe complication and may be a result of the prothrombotic nature of severe infection, systemic inflammation, and neurotropism.^15-20^

The objective of this study is to describe the neurologic and radiographic findings in a cohort of patients from a single, multi-state, multi-hospital healthcare system within the United States who tested positive for SARS-CoV-2 and underwent head imaging, with a particular focus on those with acute cerebrovascular manifestations.

## Methods

The Mayo Clinic Neurological, vascular and neurovascular Events With SARS-CoV-2 (MC NEWS) Study (IRB No. 20-003457) is a retrospective, observational study of all patients with positive SARS-CoV-2 PCR or serology or otherwise diagnosed with COVID-19 infection from 3/11/2020-7/23/2020 identified within the major campuses of Mayo Clinic and the Mayo Clinic Health System, which includes hospitals in Arizona, Florida, Minnesota, Wisconsin, and Iowa. Using the shared electronic medical record (Epic; Verona, WI), we utilized the registry of all patients with positive SARS-CoV-2 PCR or serology as well as those with recorded diagnosis of COVID-19 in the electronic medical record. This registry is an automatically generated report through the electronic medical record that tracks patients who fit the following criteria: active COVID-19 infection status, positive lab status, or diagnosis of COVID-19 on the problem list. It is continuously monitored and updated by Mayo Clinic personnel. As this registry was being developed in tandem with our data collection in the early stages, given the novel nature of the disease, we cross-validated this report with manually updated registries of patients with COVID-19 across the Mayo Clinic sites.

Because of the heterogeneity of SARS-CoV-2 infection and prior reports demonstrating neurologic manifestations as both parainfectious and postinfectious entities,^17,21,22^ we analyzed all SARS-CoV-2 patients with head imaging within 30 days of diagnosis.

For the first 3,562 patients, each chart was individually reviewed for head CT or MRI performed for any clinical indication within 30 days before or after the positive SARS-CoV-2 test. In the subsequent 5,113 patients in the registry, Mayo Clinic’s clinical data repository was queried for head imaging including CT head without contrast, CT head with contrast, CT angiogram of the head and/or neck, MRI brain without contrast, MRI brain with contrast, MR angiogram of the brain and/or neck. The reason for the change in the method of data collection was that the sheer volume of patients with positive SARS-CoV-2 tests far exceeded our expectations at the start of this project, and we required an automated process to provide a timely data analysis and dissemination of results. For the entire cohort, the number of deceased patients was also collected from the data repository.

In patients who had head imaging within a time window of ± 30 days, charts were manually reviewed and the following data were abstracted: age, sex, reason for SARS-CoV-2 testing, neurologic symptoms, past neurologic history, reason for imaging, type of imaging, imaging findings, hospital course, and disposition. Past neurologic history was manually abstracted from the electronic medical record by a physician review of clinical notes and the presence or absence of the following conditions was noted: neurodegenerative disease (including but not limited to Alzheimer’s dementia and Parkinson’s disease), prior ischemic or hemorrhagic stroke, brain tumor (primary or metastatic), epilepsy (focal or generalized), traumatic brain injury, demyelinating conditions (including but not limited to multiple sclerosis), chronic migraine or other chronic headache diagnosis. In those with cerebrovascular events, further data including location of stroke, stroke subtype, using Trial of Org 10172 in Acute Stroke Treatment (TOAST) Criteria,^23^ and laboratory markers was obtained.

**Figure 1.**
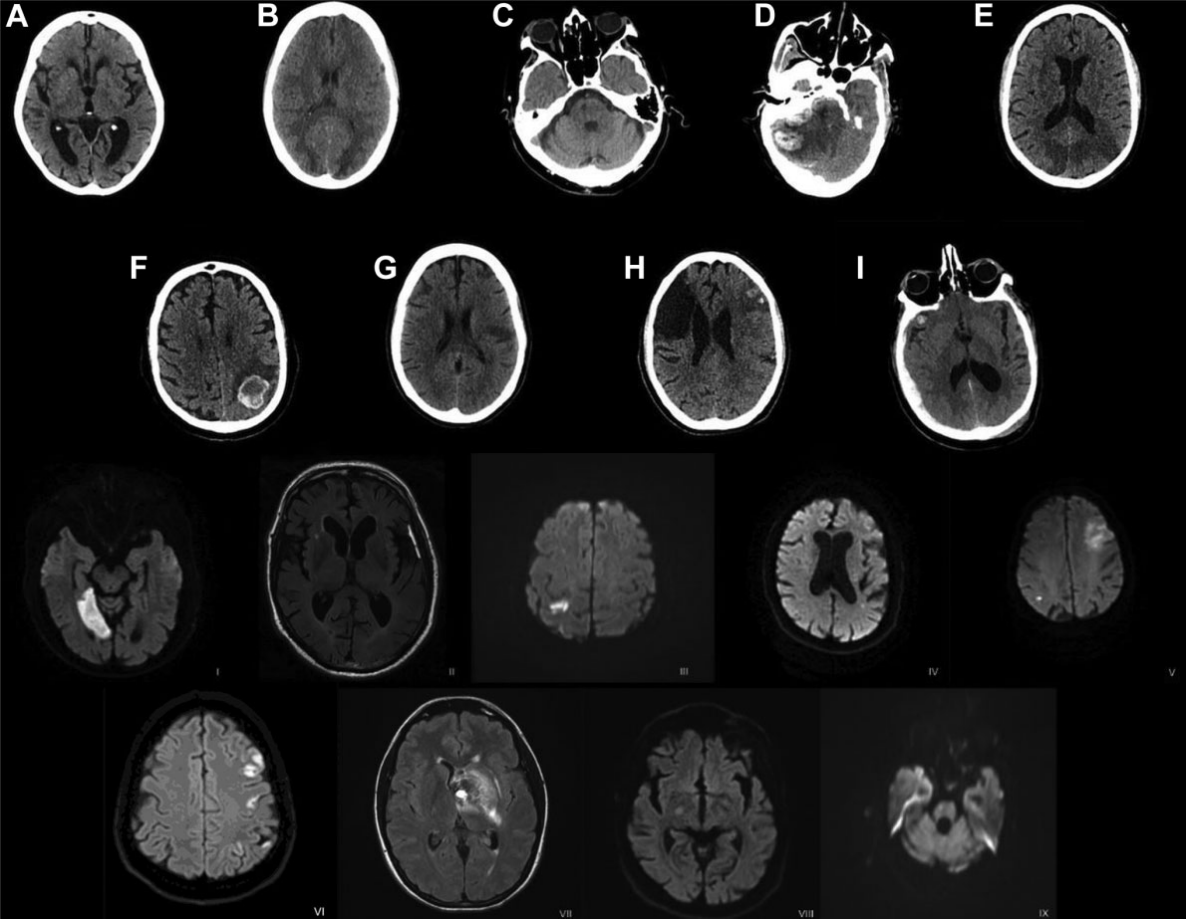
Most representative image from each of the 18 patients with SARS-CoV-2 and acute or subacute cerebrovascular events. A-I are representative CT images; I-IX are representative MRI images. (A) Subacute lacunar infarct within left basal ganglia; (B) Bi-occipital multifocal subcortical white matter hypoattenuation with gray-white junction blurring; (C) Subacute/chronic lacunar infarct in the right paramedian pons; (D) Acute large right cerebellar intraparenchymal hemorrhage with associated mass effect; (E) Acute infarcts involving the right PCA territory and left PCA/MCA border zone; (F) Multifocal intraparenchymal hemorrhage; (G) Acute/subacute infarct in the left posterior frontal lobe; (H) Acute left frontotemporal infarct with asymptomatic hemorrhagic conversion. Old right frontoparietal infarct with encephalomalacia; (I) Acute subdural hematoma overlying right lateral cerebral hemisphere with mass effect and intraparenchymal hemorrhage. (I) Acute right medial occipital infarct; (II) Thin hyperintense subdural hematoma involving the left frontotemporal region; (III) Mult-focal acute/subacute infarct within the right frontoparietal lobes; (IV) Acute left frontoparietal lacunar infarct; (V) Acute infarcts of the bilateral cerebral hemispheres; (VI) Multi-focal acute/subacute cortical infarcts in the left MCA territory (VII) Acute hemorrhagic cavernous malformation with associated developmental venous anomaly centered in the left hypothalamic region; (VIII) Small acute/subacute infarct in the posterior limb of the right internal capsule; (IX) Subacute infarct in right midbrain. (SARS-CoV-2 = severe acute respiratory syndrome coronavirus 2, CT = computed tomography, MRI = magnetic resonance imaging, PCA = posterior cerebral artery, MCA = middle cerebral artery).

The study team reviewed imaging in a 2-step manner. First, imaging reports were reviewed and classified as either acute/subacute or chronic based on the final radiology report. For those with acute/subacute imaging findings by report, digital imaging files were reviewed by the authors (MRFG, AS, and JFM) to confirm, classify, and localize findings. For those with cerebrovascular events, images were reviewed by authors (MRFG, AS, and JFM) to confirm location. A representative image from each scan is shown in Figure 1.

Severity of COVID-19 infection was defined as asymptomatic, non-severe, or severe by American Thoracic Society/Infectious Diseases Society of America criteria.^24^ Modified Rankin Scale was either extracted directly from the electronic medical record, or calculated by the certified authors based on electronic medical record documentation. National Institutes of Health Stroke Scale was extracted from the electronic medical record, where documented.

This study was approved by the Mayo Clinic COVID-19 Research Task Force and Mayo Clinic Institutional Review Board and exempted from requiring informed consent.

### Data Analysis

Continuous variables were summarized using mean with standard deviation or median with range, and categorical variables were summarized using frequency and percent. Chi-square and student’s t-test were performed to compare the baseline characteristics of MC NEWS versus imaging cohorts. Data analysis was performed using Stata (StataCorp; College Station, TX).

## Results

The first 8,675 patients who tested positive for SARS-CoV-2 from 3/11/2020-7/23/2020 were reviewed for head imaging. Of those, 180 patients underwent one or more head imaging modalities (2.07%). Patients with head imaging were older than patients without head imaging (mean age: 62 ± 19 vs. 42 ± 18 years; *p* < .001). The percentage of female patients was similar between groups with and without head imaging (76, 42.4% vs. 4,257, 49.1%; *p* = .071). Table 1 shows the demographics and baseline characteristics for each of the 180 patients. By location, 42 patients were at Mayo Clinic Rochester, 44 within the Mayo Clinic Health System, 65 at Mayo Clinic Arizona, and 29 at Mayo Clinic Florida.

**Table 1.** Baseline Characteristics of Ambulatory and Hospitalized Patients With COVID-19 and Head Imaging (N = 180).

Baseline characteristics, n (%)	Total, N = 180	Ambulatory, N = 51 (28.3)	Hospitalized, N = 129 (71.7)	P-value
Female	76 (42.2)	22 (43.1)	54 (41.9)	0.876
Age, mean ± SD Median (range), years	62 ± 19 64 (20-94)	54 ± 19 51 (21-93)	66 ± 18 69 (20-94)	0.0002
Enrollment site, n (%)				0.465
Mayo Clinic Health System	44 (24.4)	14 (27.5)	30 (23.3)	
Mayo Clinic Rochester	42 (23.3)	8 (15.7)	34 (26.4)	
Mayo Clinic Arizona	65 (36.1)	21 (36.1)	44 (16.1)	
Mayo Clinic Florida	29 (16.1)	8 (15.7)	21 (16.3)	
Prior neurologic history, n (%)				
None	107 (59.4)	39 (76.5)	68 (52.7)	0.003
Prior stroke	26 (14.4)	3 (5.9)	23 (17.8)	0.040
Neurodegenerative disease	24 (13.3)	0 (0.0)	24 (18.6)	0.001
Chronic migraine or headache	12 (6.7)	5 (9.8)	7 (5.4)	0.289
Brain tumor	6 (3.3)	1 (2.0)	5 (3.9)	0.519
Epilepsy	5 (2.8)	1 (2.0)	4 (3.1)	0.675
Traumatic brain injury	3 (1.7)	1 (2.0)	2 (1.6)	0.846
Demyelinating disease	2 (1.1)	2 (3.9)	0 (0.0)	0.024

Of the 180 patients with head imaging, 129 (71.7%) were hospitalized. Compared with the ambulatory patients with head imaging, the hospitalized patients with head imaging were older (54 ± 19 vs. 66 ± 18 years; *p* < .002). The majority of patients did not have prior neurologic history, 107 (59.4%); however, 26 (14.4%) had prior stroke, 24 (13.3%) had a history of neurodegenerative disease, 12 (6.7%) had history of chronic migraine or chronic tension type headache, 6 (3.3%) had a brain tumor, either benign or malignant, 5 (2.8%) had epilepsy, 3 (1.7%) had CNS demyelinating disease, and 2 (1.1%) had a history of traumatic brain injury.

On initial presentation to the clinic or emergency department, or while hospitalized, patients who ultimately required head imaging experienced diverse neurologic symptoms. The most common symptoms documented were encephalopathy in 72 (40.0%) and headache in 69 (38.3%). Focal neurologic symptoms including weakness, numbness, ataxia, or cranial nerve deficits were recorded in 34 patients (18.9%) and dysarthria or dysphagia was present in 8 (4.4%). Generalized weakness was reported by 20 (11.1%) patients. Syncope or presyncope was present in 19 (10.6%) patients. Ten (5.6%) patients had documented anosmia or dysgeusia. Seizure was a complicating factor in seven (3.9%) patients. In 17 (9.4%) patients, no neurologic symptom was described.

Indications for head imaging included headache for 62 patients (34.4%), encephalopathy for 61 (33.9%), focal neurologic symptoms for 30 (16.7%), trauma for 25 (13.9%), pre-operative screening for 8 (4.4%) and seizure for 2 (1.1%) (Table 2). Most patients received CT head—135 (75%), while 23 (12.8%), received brain MRI, and 22 (12.2%) underwent both modalities of head imaging.

**Table 2.** Indications for and Findings of Head Imaging (n = 180).

Head imaging	Total (n = 180)	CT only (n = 135)	MRI only (n = 23)	CT and MRI (n = 22)
Indications, n (%)				
Headache	62 (34.4)	50 (37.0)	7 (30.4)	5 (22.7)
Encephalopathy	61 (33.9)	49 (36.3)	5 (21.7)	8 (36.4)
Focal Neurologic Symptom	30 (16.7)	16 (11.9)	7 (30.4)	7 (31.8)
Trauma	25 (13.9)	20 (18.5)	0 (0.0)	0 (0.0)
Pre-Operative Screening	8 (4.4)	1 (0.7)	2 (8.7)	2 (9.1)
Seizure	2 (1.1)	1 (0.7)	1 (4.4)	0 (0.0)
Acute findings, n (%)	
No acute findings	155 (86.1)	125 (92.6)	15 (65.2)	15 (68.2)
Acute infarct	8 (4.0)	3 (2.2)	2 (8.7)	3 (13.6)
Acute intracranial hemorrhage	6 (3.3)	4 (3.0)	2 (8.7)	0 (0.0)
Subacute infarct	5 (2.8)	2 (1.5)	1 (4.4)	2 (9.1)
Posterior reversible encephalopathy syndrome	1 (0.6)	1 (0.7)	0 (0.0)	0 (0.0)
Other	7 (3.9)	2 (1.5)	3 (13.0)	2 (9.1)
Chronic findings, n (%)				
No chronic findings	99 (55.0)	75 (55.6)	14 (60.9)	10 (45.5)
Chronic infarct	33 (18.3)	24 (17.8)	3 (13.0)	6 (27.3)
Small vessel disease	43 (23.9)	32 (23.7)	3 (13.0)	8 (36.4)
Mass lesion	3 (1.7)	2 (1.5)	0 (0.0)	1 (4.6)
Atrophy, out of proportion to age	11 (6.1)	9 (6.7)	0 (0.0)	2 (9.1)
Demyelinating disease	3 (1.7)	2 (1.5)	0 (0.0)	1 (4.6)

In patients who underwent head imaging, the majority were ultimately discharged home (118, 65.6%). Other discharge locations included skilled nursing facility, long term care facility, or transitional care unit (28, 15.6%), acute inpatient rehabilitation (8, 4.4%), hospice (5, 2.8%). Four (2.2%) patients were still admitted at the time of this manuscript. Seventeen patients (9.4%) were deceased at the time of discharge. By comparison, in the overall cohort of 8,675 patients, 118 (1.4%) were deceased at the time of this manuscript.

Cerebrovascular events were the primary imaging focus of the study and defined as acute infarct, acute intracranial hemorrhage, subacute infarct, and posterior reversible encephalopathy syndrome (PRES). Of the 180 patients with head imaging, 18 (10.0%) had an acute or subacute cerebrovascular event. When compared with the original cohort of 8,675, 0.21% had and acute or subacute cerebrovascular event. Eight patients had diagnosis of acute ischemic stroke, six had acute intracranial hemorrhage (subdural hematoma, subarachnoid hemorrhage, and intraparenchymal hemorrhage—including two hemorrhagic transformations of ischemic stroke), five had subacute stroke, and one had PRES. Seven patients had other imaging findings including demyelinating lesions, mass lesions, and post-operative changes. The remaining 156 (86.7%) had no acute intracranial findings. Representative images from the patients with acute or subacute cerebrovascular events are shown in Figure 1. Evidence of chronic small vessel disease, including subcortical hypodensities, enlarged perivascular spaces, and chronic lacunar infarcts, were seen in 29 (16.1%) patients.

Table 3 describes the eighteen patients with acute or subacute cerebrovascular events in detail. Within this group, the severity of SARS-CoV-2 presentation varied. Five patients were asymptomatic, 5 had non-severe symptoms consistent with COVID-19, and 8 had severe pneumonia, as characterized by the American Thoracic Society/Infectious Diseases Society of America criteria.^24^ In the 18 patients with acute or subacute cerebrovascular events, modified Rankin scale score at the time of discharge was 0-2 in 8 (44.4%) and 3-6 in 10 (55.6%). At the time of discharge, 5 (27.8%) of the 18 patients with acute or subacute cerebrovascular events were either deceased or discharged to hospice. Of the 5 patients who were either deceased or discharged to hospice, 4 had severe pneumonia by IDSA/ACS criteria, and 1 was asymptomatic from a pulmonary perspective.

**Table 3.** Description of Patients With Acute or Subacute Cerebrovascular Events (n = 18).^1^

	Number/sample size (%)
Severity of COVID-19 illness	
Asymptomatic	5/18 (27.8)
Non-severe	5/18 (27.8)
Severe	8/18 (44.4)
Modified Rankin Scale at discharge	
0-2	8/18 (44.4)
3-6	10/18 (55.6)
Laboratory Characteristics (mean, median, range)	
White blood cell count, initial (normal, 3.4-9.6 x 10(9)/L)	7.84, 7.55, (2.8-16.8)
Platelet, initial (normal, 135-317 x10(9)/L)	212.39, 201 (48-406)
Ferritin, peak (n = 14) (normal, 24-337 mcg/L)	627.64, 531 (126-1762)
D-dimer, initial (n = 14) (normal ≤500 ng/mL FEU)	3,391.64, 1,399 (472-18,370)
D-dimer, peak (n = 14) (normal ≤500 ng/mL FEU)	10,770, 3,728.5 (595- >4200)
Chronic cerebral small vessel disease	11/18 (61.1)
Acute or Subacute Ischemic Stroke	13/18 (44.4)
Stroke Mechanism	
Unknown cause (cryptogenic)	6/13 (46.2)
Other defined cause	3/13 (23.1)
Small vessel	2/13 (15.4)
Cardioembolic	1/13 (7.7)
Large vessel	1/13 (7.7)
Multiple territories	6/13 (46.2)
National Institutes of Health Stroke Scale (n = 8)	
0-5	7/8 (86.0)
6-13	0/8 (0)
14 and higher	1/8 (12.5)
Acute Reperfusion Therapy	
IV thrombolysis	0/18 (0)
Mechanical reperfusion	1/18 (5.6)
Intracranial Hemorrhage (n = 6)	6/18 (33.3)
Intracerebral	5/6 (83.3)
Hemorrhagic conversion of ischemic stroke	2/5 (40.0)
Nontraumatic primary intraparenchymal hemorrhage	1/5 (20.0)
Traumatic primary intraparenchymal hemorrhage	1/5 (20.0)
Cavernoma	1/5 20.0)
Subarachnoid	1/6 (16.6)
Traumatic	1/1 (100)
Subdural	2/6 (33.3)
Traumatic	2/2 (100)

^1^Includes acute or subacute ischemic stroke, intracranial hemorrhage, and posterior reversible encephalopathy syndrome.

In those with acute or subacute ischemic stroke (13 patients), the most common stroke mechanism per TOAST criteria was unknown or cryptogenic in 6 (46.2%), followed by other determined causes in 3 (23.1%), small vessel in 2 (15.4%), large vessel in 1 (7.7%), and cardioembolic in 1 (7.7%). Of the other determined causes, two were thought to have hypercoagulable states from either COVID-19 infection or malignancy, and one patient had an in-stent thrombosis in the setting of anti-platelet medication noncompliance. Within this group, 6 (46.2%) had stroke within multiple vascular territories. Only one patient was eligible for and underwent acute reperfusion therapy with mechanical thrombectomy of the in-stent thrombosis.

Intracranial hemorrhages included intraparenchymal, subarachnoid, and subdural and occurred in six patients. Trauma was the most common cause of hemorrhage. Two patients had hemorrhagic conversion of ischemic stroke. One patient had multicompartment spontaneous intracranial hemorrhage, and one patient had a cavernoma with hemorrhage.

**Figure 2.**
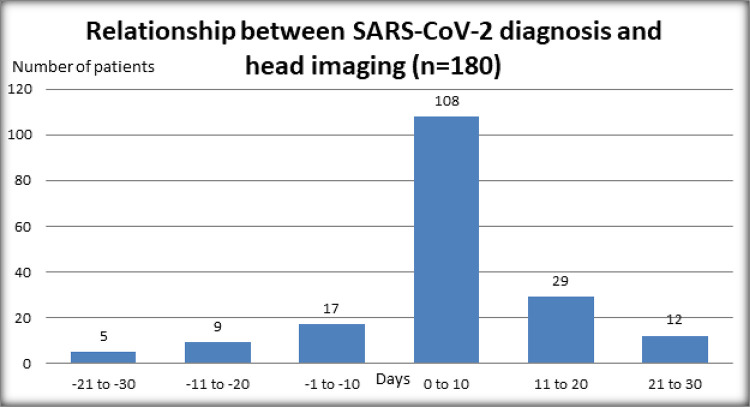
Relationship between the date of SARS-CoV-2 diagnosis and date of head imaging in 180 SARS-CoV-2 patients with head imaging.

The relationship between the time of SARS-CoV-2 diagnosis and date of head imaging are displayed in Figure 2. This figure highlights the majority of patients—125 (69.4%)—received head imaging on the same day or within 10 days of diagnosis. Of the 18 patients with acute or subacute cerebrovascular events, 11 had imaging within 10 days of SARS-CoV-2 diagnosis. Only one patient with a cerebrovascular event had imaging prior to SARS-CoV-2 positive test, and this patient was asymptomatic from SARS-CoV-2. Of the 17 patients with cerebrovascular events who had imaging on the day of diagnosis or after SARS-CoV-2 diagnosis, 11 had imaging within 10 days. Of the six patients who had imaging more than 10 days after SARS-CoV-2 diagnosis and had a cerebrovascular event (a range of 13-29 days after diagnosis), five had severe COVID-19 pneumonia. In one case, imaging was performed 29 days after diagnosis because the patient was found to have unilateral weakness after extubation and a subacute ischemic stroke was diagnosed.

## Discussion

This retrospective review of 8,675 consecutive patients with SARS-CoV-2 within a single multi-center institution identified 180 (2.07%) patients who received head imaging within 30 days of SARS-CoV-2 diagnosis, 18 (0.21%) of whom had an acute or subacute cerebrovascular event. Patients who underwent head imaging were significantly older than the overall cohort (mean 62 years old compared with mean 42 years old, *p* < .001).

This institution has taken an aggressive approach to screening patients in the hospital and clinic for SARS-CoV-2. Institutional protocol at the time of this manuscript includes PCR testing for SARS-CoV-2 in any patient with new fever or symptoms including cough, shortness of breath, sore throat, diarrhea, respiratory distress, chills, myalgias, loss of smell, change or loss of taste; all patients admitted to the hospital; and pre-operative screening, regardless of symptoms.

In comparison with other published studies, our rate of cerebrovascular events is lower (0.21%, compared with 0.9%-5.7%).^2,3,16,18,21,25^ While the reasons for this low rate of cerebrovascular events are not clear, this likely represents the heterogeneity of our cohort and oversampling of asymptomatic patients. Notably, our series includes patients across five states in multiple regions of the United States (Southeast, Southwest, Midwest) with a broad spectrum of infection from asymptomatic ambulatory patients to critically ill patients. Prior studies have focused mainly on critically ill hospitalized patients or those who presented to the emergency department and not ambulatory patients, with the exception of one study of 40,469 patients from a global de-identified database which included inpatients and outpatients and defined a rate of stroke and transient ischemic attack (TIA) of 1% based on ICD-10 codes.^3^ It is possible that patients with acute ischemic stroke and SARS-CoV-2 did not present to the hospital, as institutions have reported decreased volumes of stroke patients.^26,27^ Another consideration is that patients with severe, disabling strokes may have died before caregivers appreciated an indication for head imaging.

Interestingly, while previous studies found a higher rate of cerebrovascular events in critically ill patients,^2,22^ there was no apparent association between severity of COVID-19 illness and the likelihood of a cerebrovascular event in our cohort (5, 27.8% asymptomatic, 5, 27.8% non-severe, 8, 44.4% severe). In one study of patients from Wuhan, China, 78 patients (36.4%) had neurologic manifestations. Of those, patients with more severe infection were more likely to have cerebrovascular disease (5, 5.7% compared with 1, 0.8%).^2^ In another study from New York, 81.3% (26 of 32 patients) in their cohort of patients admitted with ischemic stroke and COVID-19 met criteria for severe infection.^22^ For example, the only patient in our cohort who underwent mechanical thrombectomy for acute ischemic stroke remained asymptomatic from his COVID-19 infection throughout the duration of his hospitalization. It is possible that the broad testing criteria within our hospital system has allowed for a higher number of asymptomatic or mildly symptomatic patients to be identified concurrently with neurologic symptoms that led to hospitalization. An alternative explanation is that the SARS-CoV-2 variant within our population is genetically distinct from other cohorts, as recent studies suggest a more mild infection in patients with certain SARS-CoV-2 variants.^28^

In patients with ischemic stroke in this cohort, the rate of cryptogenic stroke (6, 46.2%) was higher than previously published rates of 20-30%^29^ in the general population, but similar to other series of patients with SARS-CoV-2, 35% and 65.6%, respectively.^16,22^ It is possible that the increased rate of cryptogenic stroke in SARS-CoV-2 patients is a manifestation of the hypercoagulability and hyperinflammatory state known to accompany severe COVID-19.^5^ Another potential explanation for the increase in measured rate of cryptogenic stroke diagnosis may be increased diagnostic uncertainty by providers, given the unknowns of COVID-19. Additionally, as pointed out previously,^22^ patients may have an undiagnosed cause of stroke, not clearly elucidated by the time of the data collection, as stroke workup typically requires prolonged cardiac monitoring and other testing that may take weeks to complete.

Indications for head imaging were most often encephalopathy or headache, which, when combined, comprised 68.3% of all indications for head imaging. The majority of head imaging was negative for acute intracranial findings (86.1%). The pathophysiology of nonspecific central nervous system symptoms, like headache and encephalopathy, may relate to neurotropic spread of the virus via the angiotensin converting enzyme 2 (ACE2) receptor leading to a proinflammatory cascade and cytokine activation.^5^ This hyperinflammatory state has been associated with steroid responsive encephalopathy and negative imaging findings.^30^ Additional factors include hypoxia, metabolic derangements, and medications as part of the viral syndrome.^5,14,31^

Within the United States, there have been 199,606 deaths and 6,819,651 cases as of September 21, 2020, for a mortality rate of 2.93%. Within our overall cohort of 8,675 patients, 118 had died at the time of this publication, with a rate of 1.36%. Seventeen of the 180 patients who underwent head imaging were deceased at discharge, 9.44%. Five (27%) of the 18 patients who had cerebrovascular events were either deceased or discharged to hospice. Of those patients, four had severe COVID-19 pneumonia, and one was asymptomatic. The older age of patients who underwent head imaging may be a contributing factor to the higher rate of death at discharge, as well as other comorbidities and the indications for head imaging.

One major limitation of this study is the retrospective nature of data collection. By retrospectively analyzing patients with concurrent SARS-CoV-2 and head imaging, cases too mild to present for medical attention or too severe to survive to imaging were likely missed. Because our institution has been aggressive in testing patients, our proportion of asymptomatic cases is likely higher than most other centers, which could contribute to our lower rate of acute or subacute cerebrovascular events. It is, therefore, hard to know the true prevalence of SARS-CoV-2 related cerebrovascular events relative to coincidental infection. Our low number of patients with acute/subacute head imaging findings and acute/subacute cerebrovascular events is a limitation, particularly as it pertains to statistical analysis, and should be interpreted with caution. Additionally, because of the rapidly changing landscape, some of the patients in this study are still undergoing diagnostic testing and treatment, which could change their eventual diagnosis or outcome.

In conclusion, in ambulatory and hospitalized patients with SARS-CoV-2, the rate of head imaging is low, and most often performed for indication of encephalopathy and headache. Cerebrovascular events rarely occurred and were variable in acuity, severity, and mechanism. Further long-term, prospective study is needed to more clearly define the spectrum and frequency of SARS-CoV-2-related neurologic disease.
